# Health-Promoting Effects of *Thymus herba-barona*, *Thymus pseudolanuginosus*, and *Thymus caespititius* Decoctions

**DOI:** 10.3390/ijms18091879

**Published:** 2017-08-31

**Authors:** Andrea F. Afonso, Olívia R. Pereira, Rodrigo T. Neto, Artur M. S. Silva, Susana M. Cardoso

**Affiliations:** 1 Department of Chemistry & QOPNA, University of Aveiro, 3810-193 Aveiro, Portugal; andrea.afonso@ulsne.min-saude.pt (A.F.A.); rodrigotrepaneto@gmail.com (R.T.N.); artur.silva@ua.pt (A.M.S.S.); 2 Public Health Laboratory of Bragança, Local Health Unit, Rua Eng. Adelino Amaro da Costa, 5300-146 Bragança, Portugal; 3 Department of Diagnostic and Therapeutic Technologies, School of Health Sciences, Polytechnic Institute of Bragança, Av. D. Afonso V, 5300-121 Bragança, Portugal; oliviapereira@ipb.pt

**Keywords:** thymus, thyme, LC-MS, mass spectrometry, phenolic, antioxidant, anti-inflammatory, antiradicalar, antimicrobial activity

## Abstract

*Thymus herba-barona*, *Thymus pseudolanuginosus*, and *Thymus caespititius* decoctions were screened for their phenolic constituents, along with their potential antioxidant, anti-inflammatory, and antibacterial activities. The total phenolic compounds in the extracts of the three plants ranged from 236.0 ± 26.6 mgGAE/g (*T. caespititus*) to 293.0 ± 30.5 mgGAE/g of extract (*T. pseudolanuginosus*), being particularly rich in caffeic acid derivatives, namely rosmarinic acid and its structural isomers, as well as flavones, such as luteolin-*O*-glucuronide. The *T. pseudolanuginosus* extract presented the best DPPH radical scavenging ability (EC_50_ = 10.9 ± 0.7 µg/mL), a high reducing power (EC_50_ = 32.2 ± 8.2 µg/mL), and effectively inhibited the oxidation of β-carotene (EC_50_ = 2.4 ± 0.2 µg/mL). The extracts also showed NO^●^ scavenging activity close to that of ascorbic acid, and thus might be useful as anti-inflammatory agents. In addition, they exhibited antibacterial activity against gram-negative and gram-positive bacteria. *Staphylococcus aureus* strains were the most sensitive bacteria to thyme extracts, with minimum inhibitory concentration and minimum bactericidal concentration values in the range of 0.6–3.5 mg/mL. Overall, this work is an important contribution for the phytochemical characterization and the potential antioxidant, anti-inflammatory, and antimicrobial activities of these three *Thymus* species, which have been poorly explored.

## 1. Introduction

The *Thymus* genus encloses about 350 species, which are particularly well adapted to the hot and dry climate of the Mediterranean region and widespread in the arid parts of the Iberian Peninsula [1,2]. As for the vast majority of Lamiaceae plants, *Thymus* are recognized as being strongly aromatic and are widely used as spices to enhance sensory attributes such as the taste and aroma of foods [1]. These properties highlight the potential application of *Thymus* plants in the food industry, e.g., in meat, butter, chewing gum, liqueurs, ice cream, and candy production. Besides flavouring, their usage also improves the preservation of food, contributing to the prevention of food oxidation and color changes [3,4]. Likewise, these plants have some potential applications in pharmaceutical and cosmetic industries due to their biological and medicinal benefits. They are largely used in the manufacturing of perfumes, pharmaceutical products, and toilet articles [4,5,6,7]. Although these applications have been mostly associated with essential oils, nowadays, *Thymus* polar extracts are an attractive target for the screening of health-promoting properties for possible industrial applications in food, cosmetics, or pharmaceutical industries, among others [1,8].

Several authors have demonstrated that Lamiaceae, and particularly *Thymus* plants, are rich in bioactive phytochemicals, including phenolic acids and flavonoids [1,3,9,10], which in turn, have been shown to reduce the risk of diseases due to their extensive biological properties, including those which are antioxidant, cardioprotective, anticancer, anti-ageing, anti-inflammatory, and antibacterial [5,6,8,11].

The screening of the potential bioactive effects of *Thymus* polar extracts has been mainly conducted for *Thymus vulgaris* [12,13,14,15] and *Thymus serpyllum* (wild thyme) [2,16], although other species, including *Thymus pulegioides* [4,15], *Thymus praecox* [15], *Thymus sipyleus* [17], *Thymus striatus* [15], *Thymus longicaulis* [15], *Thymus mastichina* [18], have also been studied with respect to their antioxidant activities. Overall, aqueous or alcoholic extracts from *Thymus* have been shown to exhibit strong antioxidant activities in in vitro [17,19] or in vivo models [20]. Moreover, other effects reported in the polar extracts of thyme species include antimicrobial [21,22], antidiabetic [8,19], neuroprotective [15], and inflammatory properties [23].

In contrast to the above mentioned studies, the potential applications of the polar extracts from less-widespread *Thymus* like *Thymus herba-barona*, *Thymus pseudolanuginosus*, and *Thymus caespititius* remain unexplored. These three species are particularly known for being well-adapted to the Atlantic climate, thus growing spontaneously in the Iberian Peninsula and Mediterranean islands. As reported, their essential oils exhibit antioxidant, antimicrobial, and insecticidal effects and can potentially be used as preservatives in storage products for food flavouring, and as deodorants and disinfectants [24,25]. The present study aims to contribute to the clarification of phenolic constituents of *T. herba-barona*, *T. pseudolanuginosus*, and *T. caespititius*, as well as to exploit their free radical scavenging, antibacterial, and anti-inflammatory activities.

## 2. Results and Discussion

### 2.1. Phenolic Compounds in Thymus Aqueous Extracts

The mass yield of thyme aqueous extracts ranged from 15% to 20%, with *T. caespititius* being the most predominant (Table 1). In turn, the latter showed a lower total phenolic content (236 ± 26.6 mg GAE/g extract, equivalent to 47.0 mg GAE/g dry plant) in comparison to the mean values obtained for the *T. herba-barona* and *T. pseudolanuginosus* extracts (273 and 293 mg GAE/g extract or 41.6 and 49.2 mg GAE/g dry plant, respectively). However, it is important to note that these three aqueous extracts had a considerably higher total phenolic content than those found by other authors in distinct *Thymus* plants. In particular, aqueous extracts obtained from wild thyme (*T. serpyllum*) at 50 and 100ºC were found to contain 79.02 ± 6.62 and 91.07 ± 9.25 mg GAE/g extract, respectively [16], while aqueous extracts obtained from *Thymus zygis* leaves at 20ºC produced a 25.8 ± 2.0 mg GAE/g dry sample [3]. In addition, the phenolic content in phosphate buffer or hydromethanolic extracts from *T. vulgaris* were reported to account for 2.1 ± 0.1 mg GAE/g fresh leaves [26] or 19.2 ± 0.3 mg GAE/g of fresh weight [14], respectively.

**Figure 1:**
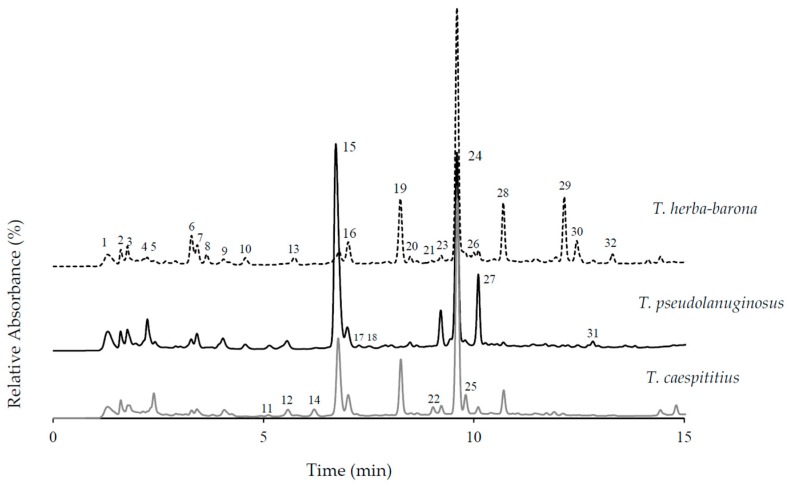
Chromatographic profiles at 280 nm of *T. herba-barona*, *T. pseudolanuginosus*, and *T. caespititius* aqueous extracts (The numbers in the figure correspond to the LC-MS^n^ fractions indicated in Table 2).

Individual phenolic constituents of the three thyme aqueous extracts were elucidated by ultra high performance liquid chromatography coupled to diode array detector and an a electrospray mass spectrometer (UHPLC-DAD-ESI-MS^n^), taking into consideration the gathered ultraviolet-visible (UV-Vis) and mass spectrometry (MS) spectra data of the eluted chromatographic peaks (Figure 1, Table 2) and in comparison to those of standard compounds and/or in comparison to literature data.

Note that despite the fact that *Thymus* plants are generally known for their richness in rosmarinic acid [1], the specific phenolic composition of thyme extracts is dependent on several factors, including the botanical species and the applied extraction conditions. To the best of our knowledge, there is no reported literature regarding the phenolic composition of *T. herba-barona*, *T. pseudolanuginosus*, and *T. caespititius* extracts.

Rosmarinic acid was a major phenolic component in the three thyme extracts, accounting for 55.8 ± 2.8 mg/g in *T. herba-barona* and 40.2 ± 0.9 and 43.2 ± 3.2 mg/g in *T. pseudolanuginosus* and *T. caespititius*, respectively. Despite the common abundance, rosmarinic acid was clearly less representative in the *T. pseudolanuginosus* extract (30% of total quantified phenolics) in comparison to the other two, in which it amounted for 45–52% of the total quantified phenolics. This difference was mainly due to the high abundance of luteolin-*O*-glucuronide (6.8 min, [M − H]^−^ at *m/z* 461→285) in the *T. pseudolanuginosus* extract, which accounted for 54.1 ± 0.6 mg/g, while its levels were only 17.3 ± 1.1 mg/g and 4.4 ± 0.02 mg/g in the *T. caespititius* and *T. herba-barona* extracts, respectively. Globally, the two *O*-glucuronide derivatives of luteolin eluted in fractions 15 and 16 (RT 6.8 and 7.0 min respectively) in the *T. pseudolanuginosus* extract accounted for 61.2 mg/g of the extract and represented 46% of its total phenolics. This caused a clear differentiation between the extracts, with *T. pseudolanuginosus* phenolic components being mainly represented by flavones. Note that *O*-glucuronide derivatives of luteolin and other flavone glycosides such as luteolin-*C*-glucoside, apigenin-*O*-glucuronide and apigenin-*O*-glucoside herein detected have also been previously described for other *Thymus* species, but still, their levels, particularly those of luteolin-*O*-glucuronide derivatives, are higher in *T. pseudolanuginosus* than in previously reported data (8–14 mg/g of dry plant) [1,17,27].

Apart from rosmarinic acid, the remaining caffeic acid derivatives represented 8%, 13%, and 34% of the total quantified phenolic compounds in *T. caespititius*, *T. pseudolanuginosus*, and *T. herba-barona* extracts, respectively. Among the three plant species, the latter was clearly the richest in this group of compounds, comprising simple compounds, namely caffeic acid (4.3 ± 0.1 mg/g extract) and *t*-5-*O*-CQA (in vestigial concentrations), as well as several depsides (Table 2), namely 3′-*O*-(8″-*Z*-caffeoyl)rosmarinic acid (a compound previously described in other thyme species [28]) and/or its isomers (MW 538 Da, fractions 25, 28 and 32), together with dihydro-salvianolic acid B (fraction 19; MW 716 Da). On the other hand, salvianolic acids K (fraction 27, [M − H]^−^ at *m/z* 555 → 493 → 359) and B (fraction 19, [M − H]^−^ at *m/z* 717 → 519 → 475) were found in the *T. pseudolanuginosus* and *T. caespititius* extracts, with levels of 10.5 ± 0.1 and 6.9 ± 0.5 mg/g of the extract, respectively.

### 2.2. Antioxidant Capacity

The overproduction of oxidants is responsible for the pathogenesis of many chronic diseases. In turn, distinct phytochemicals from foods and medicinal plants exhibit antioxidant properties that might be beneficial to counteract oxidative events [11]. In this context, the three *Thymus* aqueous extracts were investigated for their antioxidant abilities through distinct in vitro methods, namely DPPH^●^, reducing power and β-carotene bleaching assays. The DPPH^●^ scavenging method evaluates the free radical scavenging ability of the plant extracts to trap the synthetic free radicals DPPH^●^, while the reducing power and β-carotene bleaching methods measure the extract’s ability to reduce Fe^3+^ to Fe^2+^ or to inhibit lipidic peroxidation, respectively.

The antioxidant potentialities of *T. herba-barona*, *T. pseudolanuginosus*, and *T. caespititius* aqueous extracts are detailed in Table 1, in terms of their EC_50_ values. Considering the results, one can conclude that all three *Thymus* extracts showed a high antioxidant capacity, which was particularly evident in the DPPH^●^ and reducing power tests, for which the EC_50_ values are 1.6–2.0 higher than those of the reference commercial compounds. Among the three extracts, there is a tendency for the better activity of *T. pseudolanuginosus* (EC_50_ of 10.9 ± 0.7 and 32.2 ± 8.2 µg/mL for DPPH^●^ and reducing power tests, respectively), although differences are not statistically significant.

The high antioxidant ability herein reported is in accordance with the literature data found for *Thymus* plants. Indeed, low DPPH^●^ EC_50_ values were previously registered for *T. serpyllum* L. aqueous extracts (EC_50_ of 13.75 ± 1.14 and 11.76 ± 0.25 µg/mL for extracts obtained at 50 and 100 °C, respectively) [16] and for the ethanolic extracts from *T. longicaulis*, *T. praecox*, *T. pulegioides*, *T. serpyllum*, *T. striatus*, and *T. vulgaris*, which exhibited DPPH^●^ EC_50_ values in the range of 3.01–6.01 µg/mL, i.e., 1.8–3.6 higher than those of the reference commercial compounds [15]. Less promising results were previously reported for *Thymus capitatus* methanolic and hexane extracts, with EC_50_ = 44.5 ± 1.9 and 38.2 ± 1.2 µg/mL, respectively (eight to nine times less active than the positive control EC_50_ = 5.0 ± 0.8) [29] and for hydroalcoholic extracts from *Thymus pubescens*, *Thymus kotschyanus*, and *Thymus daenensis* (EC_50_ range between 31.47 and 48.68 µg/mL), also six to nine times less active that the positive control, i.e., galic acid [30]; for *T. vulgaris* and wild thyme infusions, the EC_50_ values were 300 and 450 µg/mL, respectively, and corresponded to a 17–25 times lower efficacy than butylated hydroxytoluene [12]. The ability of *Thymus* species to reduce Fe^3+^ to Fe^2+^ is not reported as often; however, Kindl et al. have reported a strong ability for ethanolic extracts from *T. longicaulis*, *T. praecox*, *T. pulegioides*, *T. serpyllum*, *T. striatus*, and *T. vulgaris* (EC_50_ = 11.4–15.1 µg/mL), with the best results being registered for *T. pulegioides*, whose EC_50_ was only 1.7 higher than the reference control [15].

Contrary to the results of the above antioxidant methods, the three *Thymus* extracts presented clear differences in the β-carotene bleaching assay. The oxidation of β-carotene was effectively inhibited by *T. pseudolanuginosus* (EC_50_ = 2.4 ± 0.2 µg/mL), followed by *T. caespititius* (EC_50_ = 6.1 ± 0.2 µg/mL), while *T. herba-barona* was ineffective (Table 1). Notably, literature data also point out promising activity toward the β-carotene oxidation protection from other thymes. In particular, Iauk et al. [29] described an EC_50_ value for a *T. capitatus* methanolic extract that was close to that of 0.7 ± 0.03 µg/mL in 30 min and 1.9 ± 0.6 µg/mL in 60 min. Higher values were observed for the ethanolic extracts from *T. daenensis*, *T. kotschyanus*, and *T. pubescens* (EC_50_ = 23.7, 35.2, and 92.9 µg/mL, respectively) [30].

The combined information from the three antioxidant methods, together with that from the phenolic compounds´ levels, suggests that these last constituents are not the only actors dictating the antioxidant potential of the extracts. Indeed, although phenolic compounds are abundant (particularly flavones) in the *T. pseudolanuginosus* extract, which can be correlated to its superior antioxidant ability in comparison to those of the remaining extracts, the direct phenolic content and antioxidant activity were not observed for those of the two remaining plant species in the β-carotene bleaching assay. This phenomenon could be due to the presence of oxidative—inducer components in the *T. herba-barona* extract, thus mitigating the activity of the antioxidant phenolic compounds.

### 2.3. Anti-Inflammatory Activity

Because of the major relevance of inflammatory processes in the onset of numerous diseases (cancer, Alzheimer, heart failure, ischemic stroke, and others), the search for low-toxic natural extracts able to counteract pivotal inflammatory players has increased dramatically in the last years [31,32]. The aqueous extracts from the three plant species were screened for their ability to counteract two key inflammatory events, namely the increment of NO^●^ (i.e., a main proinflammatory mediator produced by macrofages) and lipoxygenase (LOX) activity (i.e., the enzyme that controls the production of proinflammatory leukotrienes) [7,33]. As detailed in Table 1, all the extracts revealed a high NO^●^ scavenging ability. Curiously, this effect was more evident for *T. caespititius*, which exhibited the same potency as ascorbic acid (EC_50_ of 229.7 ± 21.5 µg/mL and 228.0 ± 20.7 µg/mL, respectively). Despite having a low ability to inhibit LOX activity, *T. caespetitius* was also the most relevant amongst the three samples (EC_50_ = 590.5 ± 166.3 µg/mL). Hence, the gathered results suggest that *Thymus* aqueous extracts of the selected plants might have potential applications as anti-inflammatory agents, acting through anti-radical capacities towards NO^●^. The fact that this ability is not directly associated the extracts´ phenolic content (*T. caespititius* extract was the less rich in phenolics) also suggests that non-phenolic compounds can have major roles in this action. To our knowledge, the NO^●^ scavenging ability of *Thymus* polar extracts has only been previously reported for ethanolic extracts of six *Thymus* species: *T. serpyllum* subsp. *serpyllum* (EC_50_ = 176.6 ± 8.1 µg/mL), *T. praecox* subsp. *polytrichus* (EC_50_ = 139.0 ± 5.7 µg/mL), *T. vulgaris* (EC_50_ = 97.9 ± 2.9 µg/mL), *T. striatus* (EC_50_ = 91.1 ± 5.3 µg/mL), *T. longicaulis* (EC_50_ = 71.6 ± 4.9 µg/mL), and *T. pulegioides* (EC_50_ = 69.8 ± 4.4 µg/mL) [15], which showed EC_50_ values 1.3–3.2 times higher than the tested standard compound (Trolox), while that of LOX inhibition activity was not exploited.

### 2.4. Antibacterial Activity

The resistance to antibiotics has been associated with diseases and high mortality rates [34]. Research into new antimicrobial substances, such as purified natural compounds from plants, can serve as one of the therapeutic strategies for the synthesis of new generation and alternative chemical drugs with low toxicity. In this field, phenolic compounds have been gaining magnitude [35]. The antibacterial activity of the Lamiaceae family has been extensively studied, with promising results being observed for *Thymus* essential oils [36,37], while little knowledge has been gathered regarding polar extracts from a thyme origin.

Overall, the collected data showed that, among the five tested strains (Table 3), *Staphylococcus aureus* was the most sensitive to the three *Thymus* extracts, which were able to inhibit both its growth and viability at 0.6, 1.6, and 3.5 mg/mL for *T. herba-barona*, *T. pseudolanuginosus*, and *T. caespititius*, respectively. These results are consistent with those reported for other *Thymus* species, such as in the study described by Benbelaïd et al. for a water extract of *Thymus lanceolatus*, which exhibited a minimum inhibitory concentration (MIC) value of 1.0 mg/ml for *S. aureus*, as evaluated by the broth microdilution method [22]. Results acquired using the agar dilution method showed MIC values of 0.78 mg/ml for ethanolic extracts of *Thymus caramanicus* when exposed to *S. aureus* [21].

The three *Thymus* plants also exhibited a relevant antibacterial effect against the remaining tested bacteria, although with less potency than the food preservative nisin. In detail, MIC values of 5.0 and 6.5 mg/mL were registered for *T. herba-barona* and *T. pseudolanuginosus*, respectively, against *Salmonella typhimurium*, *Staphylococcus epidermidis*, *Escherichia coli*, and *Pseudomonas aeruginosa*. Despite *T. caespititius* generally being the less active extract, its activity towards *S. epidermidis* (MIC of 3.5 mg/mL) was more effective than the remaining ones. Note that the MIC concentrations herein found are close to those previously reported for *T. vulgaris* ethanolic extracts, against the same panel of bacteria (MIC in the range 6.25–12.5 mg/mL) [21] or to that of *T. lanceolatus* aqueous extracts against *S. typhimurium* (MIC of 4.0 mg/mL) [22], although they are less effective than a *T. caramanicus* hydroethanolic extract towards *E. coli* and *P. aeruginosa* (MIC values of 1.56 mg/mL and 1.30 mg/mL, respectively) [21].

Overall, the gathered results showed that the thyme extracts were not lethal to some gram-negative bacteria (*S. typhimurium*, *E. coli*, and *P. aeruginosa*), with a high minimum bactericidal concentration (MBC) (Table 3). *T. herba-barona* is effective against *S. typhimurium*, but needs a concentration superior to 5.0 mg/mL to kill *E. coli* and *P. aeruginosa*; a concentration of 6.5 mg/mL of *T. pseudolanuginosus* was not effective against *S. typhimurium*, *E. coli*, and *P. aeruginosa*; *T. caespititius* can be lethal to *E. coli*, but required a concentration superior to 7.0 mg/mL to eliminate *S. typhimurium* and *P. aeruginosa*). Additionally, gram-negative bacteria were more resistant (MIC between 5.0 and 7.0 mg/mL) than gram-positive bacteria, particularly *S. aureus* (MIC in the range 0.6 –3.5 mg/mL). These results are in agreement with those of other authors, who tested aqueous extracts for *T. lanceolatus* (MIC = 4.0 mg/mL against *S. typhimurium* and MIC = 0.83 mg/mL against *S. aureus*) [22]. The same evidence was observed in several researches that tested oils extracted from *Thymus* plants [36,37].

## 3. Materials and Methods

### 3.1. Chemicals

Rosmarinic acid, apigenin-7-*O*-glucoside, luteolin-7-*O*-glucoside, eriodictyol-7-*O*-glucoside, luteolin-8-*C*-glucoside, quinic acid, caffeic acid, and *t*-5-*O*-caffeoylquinic acid were obtained from Extrasynthese (Genay Cedex, France). Gallic acid and nisin were obtained from Sigma Chemical Co. (St. Louis, MO, USA). Folin-Ciocalteu reagent, Na_2_CO_3_, formic acid, and ethanol were purchased from Panreac (Barcelona, Spain). *n*-hexane, methanol, and acetonitrile with HPLC purity were purchased from Lab-Scan (Lisbon, Portugal). Mueller-Hinton agar was obtained from VWR, Prolabo Chemicals, West Chester, PA, USA. Water was treated in a Direct-Q^®^ water purification system (Merck Life Science, Germany).

### 3.2. Plant Materials

*T. herba-barona*, *T. caespititus*, and *T. pseudolanuginosus* species were purchased as a mixture of flowers, leaves, and stems from Ervital (Viseu, Portugal). The plants had been cultivated under an organic regime and, after collection, their aerial parts (flowers, leaves, and stems) were dried in a ventilated incubator at 20–35 °C, for three to five days.

### 3.3. Extraction of Phenolic Compounds

Phenolic compounds were extracted by decoction, as according to Martins et al. [38], thyme phenolic compounds are efficiently recovered by this methodology. Decoction was performed according to the method described by Ferreira et al. [39], with adaptations. A total of 100 mL of distilled water was added to 5 g of plant material (0.5 mm mesh powder) and the mixture was heated and then boiled for 15 min. After extraction, the mixture was left to stand for 5 min, followed by filtration under reduced pressure through a G3 sintered plates filter. The resulting filtrated solution was concentrated in a rotary evaporator (BUCHI Labortechnik AG, Flawil, Switzerland) at 37 °C, followed by deffating with *n*-hexane (1:1 *v*/*v*). The aqueous defatted fraction was frozen, freeze-dried, and kept under vacuum in a desiccator in the dark, for subsequent use. Three extracts were obtained for each plant.

### 3.4. Identification and Quantification of Phenolic Compounds

The total phenolic content of each *Thymus* extract was determined according to the adapted Folin-Ciocalteu colorimetric method, as described by Pereira et al. [40]. The individual phenolic compounds were identified by a UHPLC-DAD-ESI-MS^n^ analysis of extracts (5 mg/mL), performed on Ultimate 3000 (Dionex Co., Sunnyvale, CA, USA) apparatus equipped with an ultimate 3000 Diode Array Detector (Dionex Co.) and coupled to a mass spectrometer. The chromatographic apparatus was composed of a quaternary pump, an autossampler, a photodiode-array detector, and an automatic thermostatic column compartment. The column used had a 100 mm length, 2.1 mm i.d., 1.9 µm particle diameter, and end-capped Hypersil Gold C18 column (Thermo Scientific, Waltham, MA, USA), and its temperature was maintained at 30 °C. Gradient elution was carried out with a mixture of 0.1% (*v*/*v*) of formic acid in water (solvent A) and acetonitrile (solvent B), which were degassed and filtered before use. The solvent gradient consisted of a series of linear gradients, starting with 15–28% of solvent B over 5.6 min, increasing to 29% at 8.8 min, 100% of solvent B at 13.1 min, and maintaining this value up to 17 min, followed by the return to the initial conditions, with a total running time of 20 min. The flow rate used was 0.2 mL·min^−1^ and the UV–Vis spectral data for all peaks were accumulated in the range 200–600 nm. The chromatographic profiles were recorded at 280, 320, and 340 nm.

The mass spectrometer used was a Thermo LTQ XL (Thermo Scientific) ion trap MS equipped with an ESI source. Control and data acquisition were carried out with the Thermo Xcalibur Qual Browser data system (Thermo Scientific). Nitrogen above 99% purity was used and the gas pressure was 520 kPa (75 psi). The instrument was operated in negative-ion mode with an ESI needle voltage set at 5.00 kV and an ESI capillary temperature of 275 °C. The full scan covered the mass range from *m/z* 100 to 2000. CID–MS/MS and MS^n^ experiments were simultaneously acquired for precursor ions using helium as the collision gas with a collision energy of 25–35 arbitrary units.

For quantitative analysis, the limits of detection (LOD) and quantification (LOQ) were calculated from the parameters of the calibration curves obtained by an injection of known concentrations of the exact or structurally-related standard compounds, represented in Table S1.

### 3.5. Bioactivity Tests

#### 3.5.1. DPPH^●^ Scavenging Test

The radical scavenging capacity of *T. herba-barona*, *T. pseudolanuginosus*, and *T. caespititius* extracts was evaluated by a DPPH radical test, according to the previously described methodology [41]. Ascorbic acid was used as the positive control.

#### 3.5.2. Reducing Power Test

The ability of *T. herba-barona*, *T. pseudolanuginosus*, and *T. caespititius* (0.05–0.25 mg/mL) aqueous extracts in reducing iron (III) was assessed by the method previously described [41]. Butylated hydroxyanisole (BHA) was used as the positive control.

#### 3.5.3. β-Carotene Bleaching Carotene

The assay was performed as previously described by Juntachote and Berghofer [42]. A stock emulsion of β-carotene/linoleic acid was initially prepared by dissolving 20 mg of β-carotene in 10 mL of chloroform. A total of 1 mL of the β-carotene solution was added to 1 g of tween 80 and, after chloroform removal, 50 mg of linoleic acid was added. Distilled water (100 mL) was added to the mixture and homogenized using the rotary evaporator. Aliquots of β-carotene/linoleic acid emulsion (250 µL) were mixed with 50 µL of extract at different concentrations and the initial absorbance at 470 nm was immediately recorded. After incubation at 50 °C for 2 h, the reaction was stopped using an ice bath and the absorbance at 470 nm was measured. The blank used was prepared by adding chloroform without β-carotene. BHA was used as the positive control. The % of inhibition was calculated using the formula:

$$\%\;\mathrm{of}\;\mathrm{inhibition}\;=\;\frac{\left(C_{t\;=\;0}-C_{t\;=\;2}\right)-\left(E_{t\;=\;0}-E_{t\;=\;2}\right)\;}{\left(C_{t\;=\;0}-C_{t\;=\;2}\right)}\times 100$$

where *C*_t=0_ corresponds to the absorbance of control at *t* = 0 min; *C*_t=2_ corresponds to the absorbance of control at *t* = 120 min; E_t=0_, Absorbance of extract at *t* = 0 min; E_t=2_, Absorbance of extract at *t* = 120 min.

#### 3.5.4. NO^●^ Scavenging Test

This assay was performed according to the method described by Bor et al. [43]. In brief, 100 µL of sodium nitroprusside (3.33 mM) in PBS 100 mM (pH = 7.4) was added to 100 µL of extract solution at different concentrations (0.07–0.5 mg/mL) and incubated for 15 min at room temperature under light irradiation. The generated NO^●^ interacts with molecular oxygen, producing NO^2−^, which in the presence of 100 µL of Griess reagent (1% of sulfanilamide and 0.1% of naphthylethylenediamine dihydrochloride in 2.5% of phosphoric acid) produces a purple azo dye. The measurement of the absorbance was determined spectrophotometrically at 562 nm and ascorbic acid was used as the positive control.

#### 3.5.5. Inhibition of 5-Lipoxygenase

The LOX inhibitory assay was performed in a quartz 96-well plate according to the Tappel et al. procedure, with some modifications [44]. During this procedure, 20 µL of the ascorbic acid or extract sample solutions and 20µL of the LOX work solution were added to each well and incubated at 37 °C in the plate reader for 10 min. After incubation, 40 µL of linoleic acid, previously heated at 37 °C, was added and quickly placed in the plate reader. The reaction was followed for 20 min taking measurements every minute at 234 nm. The reaction rate at each inhibitor concentration was calculated by determining the slope of the experimental values and the percentage of inhibition by the following formula:

$$\%\;\mathrm{of}\;\mathrm{inhibition}\;=\frac{v_{0}-v_{\left[\mathrm{inhibitor}\right]}}{v_{0}}\times 100$$

where $v_{0}$ corresponds to the reaction rate of control and $v_{\left[\mathrm{inhibitor}\right]}$ is the reaction rate of the extract.

#### 3.5.6. Antimicrobial Activity

The antibacterial potential of the *Thymus* polar extracts were evaluated against five bacterial strains, including gram-positive bacteria (*S. epidermidis* NCTC 11047 and *S. aureus* NCTC 6571) and gram-negative bacteria (*S. typhimurium* NCTC 12023, *E. coli* NCTC 9001, and *P. aeruginosa* NCTC 10662) from the National Collection of Type Cultures, operated by Public Health England. All strains were cultured in Mueller-Hinton agar and incubated at 37 °C for 24 h.

The MIC and MBC of plant extracts were determined by the broth microdilution method using a modified standard protocol [45]. Bacterial suspensions were prepared by direct colony suspensions in sterile distilled water and adjusted to obtain 1.5 × 10^8^ colony-forming units (CFU)/mL, approximately equivalent to 0.5 McFarland units. A final inoculum of 1.5 × 10^5^ CFU/mL was required for suspensions diluted in a 1:100 ratio in Mueller-Hinton broth.

One hundred microliters of Mueller-Hinton broth was dispensed into wells of 96-well micro titer plates (BioTek Instruments, Inc., Winooski, VT, USA). Aqueous solutions of *T. herba-barona*, *T. pseudolanuginosus*, and *T. caespititius* extracts were added at a final concentration of 10, 13, and 14 mg/mL, respectively, and were then serially diluted four times across the plate. One hundred microliters of bacteria suspension was finally added to each well and the plates were incubated at 37 °C for 24 h. The assay for each pathogen was repeated three times.

The MIC was defined as the lowest concentration at which visible growth was inhibited while the MBC is the lowest concentration of the tested substance which has a bactericidal effect. MBC values were determined by subculturing 10 µL of the culture from each negative well onto Mueller-Hinton agar and then determining the dilution at which growth was detected [46].

The solvent without extracts served as the negative control and nisin was used as the positive control. Nisin is an antibacterial polypeptide approved as a food preservative whose stock solution was prepared by dissolving 2 mg of nisin in 1 mL of HCl 0.02 N [47].

### 3.6. Statistical Analysis

All data are presented as mean ± standard deviations. One-way analysis of variance (ANOVA) followed by a Tukey’s test were used to detect any significant differences among different means. A *p*-value of less than 0.05 was assumed to indicate a significant difference. The results were analyzed using GraphPad Prism (GraphPad Software, CA, USA, version 6.0).

## 4. Conclusions

This work elucidates the phenolic composition of *T. herba-barona*, *T. pseudolanuginosus*, and *T. caespititius* decoctions, whilst also exploiting their antioxidant, anti-inflammatory, and antimicrobial activities. The three aqueous extracts were mainly characterized by the presence of phenolic acids and flavonoids, which are in part related to their biological activities. *T. pseudolanuginosus* presented the best antioxidant results concerning the three methods used. The selected *Thymus* plants extracts exhibited antibacterial activity against the panel of tested bacteria (*S. typhimurium*, *S. epidermidis*, *S. aureus*, *E. coli*, and *P. aeruginosa* ), especially *S. aureus* strains, which were in general the most sensitive. *T. caespititius* appears to have anti-inflammatory potential, based on its promising inhibitory activity on NO^●^ production. Bearing in mind the increasing demand for bioactive extracts of a botanical origin, this work opens a door for the expansion of the commercial exploitation of the thyme species.

## Figures and Tables

**Table 1:** Yield of extraction (%), total phenolic content (mg GAE/g of extract), and antioxidant and anti-inflammatory activities (EC_50_, µg/mL) of *T. herba-barona*, *T. pseudolanuginosus*, and *T. caespititius* aqueous extracts.

Plant Extract	Yield (%)	TPC	DPPH^●^	RP	β-Carotene	NO^●^	5-LOX
*T. herba-barona*	15.3 ± 1.80 ^a^	273 ± 16.6 ^a,c^	11.6 ± 0.90 ^a^	35.1± 4.50 ^a^	>26.70 ^a^	286 ± 32.6 ^a^	841 ± 138 ^a^
*T. pseudolanuginosus*	16.8 ± 0.90 ^a^	293 ± 30.5 ^a^	10.9 ± 0.70 ^a^	32.2± 8.20 ^a^	2.40 ± 0.20 ^b^	299 ± 23.4 ^a^	814 ± 87.2 ^a^
*T. caespititius*	19.9 ± 2.40 ^a^	236 ± 26.6 ^b,c^	13.8 ± 0.60 ^a^	39.3 ± 2.70 ^a^	6.10 ± 0.20 ^c^	230 ± 21.5 ^b^	591 ± 166 ^a^
AA			6.70 ± 0.70 ^b^	−		228 ± 20.7 ^b^	7.80 ± 1.00 ^b^
BHA			−	16.0 ± 2.00 ^b^	0.40 ± 0.02 ^d^		

Mean values ± standard deviations. Statistical analysis was performed by one-way ANOVA, followed by a Tukey test. In each column, different letters (a–d) stand for significant statistical different data (*p* < 0.05). TPC: Total Phenolic Compounds; RP: Reducing Power; AA: Ascorbic acid; BHA: Butylated hydroxyanisole; LOX: lipoxygenase.

**Table 2:** Identification and quantification of UHPLC (ultra high performance chromatography) eluting fractions by UHPLC-DAD-MS^n^ of *T. herba-barona*, *T. pseudolanuginosus*, and *T. caespititius* aqueous extracts.

Fraction	RT (min)	λmax (nm)	Compound	[M-H]^−^	Main Fragments ESI-MS^n^	(mg/g Extract)		
						*T. h-b*	*T. pseud*	*T. caesp*
1	1.3	270	Quinic acid ^A^	191	MS2 [191]: 111, 173	D	D	D
2	1.6	278	Syringic acid-*O*-hex ^B^	359	MS2 [359]: 197, 179, 161, 153, 135	D	D	D
3	1.8	281	Danshensu ^B^	197	MS2 [197]: 179	D	D	D
		292, 323	4-*O*-CQA ^B^	353	MS2 [353]: 173, 179, 191	−	D	D
4	2.2	286, 322	*t*-5*-O*-CQA ^A^	353	MS2 [353]: 191 , 179, 161, 135, 119	D	6.4 ± 0.4	D
5	2.4	271, 333	Apigenin di-*C*-glc ^B^	593	MS2 [539]: 473, 353, 383, 503, 575, 297	D	D	4.0 ± 0.2
6	3.3	289, 321	Caffeic acid ^A^	179	MS2 [179]: 135, 151, 161, 107, 97	4.3 ± 0.1	D	D
7	3.4	287, 318	SA F der ^B^	375	MS2 [375]: 313, 269, 179, 135, MS3 [313]: 269, 161	D	D	D
		277	RA der ^B^	377	MS2 [377]: 359; MS3 [359]: 161, 179, 197, 223, 133	−	−	D
8	3.7	283	Eriodictyol-*O*-glc ^A^	449	MS2 [449]: 287, 269, 259, 267	1.9 ± 0.01	−	−
9	4.1	281, 342	Quercetin*-O*-glcA ^B^	477	MS2 [477]: 301, 343, 397	2.3 ± 0.1	3.4 ± 0.04	1.1 ± 0.08
10	4.6	341	Luteolin-*C*-glc ^A^	447	MS2 [447]: 357, 285, 327	5.1 ± 0.1	2.9 ± 0.02	−
11	5.1	282	RA sulfate ^B^	439	MS2 [439]: 259, 421, 225, 371, 359, 197; MS3 [259]: 161	−	−	D
12	5.6	253, 287, 312	SA I ^B^	537	MS2 [537]: 339, 493; MS3 [339]: 295, 229, 293	−	D	D
13	5.7	289, 318	SA F der ^B^	519	MS2 [519]: 475, 313; MS3 [475]: 313, 269, 179, 431	D	−	−
14	6.2	254, 266, 345	Luteolin-*O*-rut ^B^	593	MS2 [593]: 285	−	−	2.2 ± 0.1
15	6.8	281, 331	Luteolin-*O*-glcA (isom 1) ^B^	461	MS2 [461]: 285, 175; MS3 [285]: 267, 239, 241, 213, 185	4.4 ± 0.02	54.1 ± 0.6	17.3 ± 1.1
16	7.0	255, 265, 345	Luteolin-*O*-glcA (isom 2) ^B^	461	MS2 [461]: 285; MS3 [285]: 241, 199, 175, 151, 267	10.5 ± 0.2	7.1 ± 0.2	6.8 ± 0.4
17	7.3	261, 331	Apigenin-*O*-glc (isom 1) ^A^	431	MS2 [431]: 269	−	0.9 ± 0.15	−
18	8.0	285, 333	SA C der ^B^	553	MS2 [553]: 491, 399, 179, 429, 473; MS3 [491]: 473	−	D	−
19	8.3	254, 283, 344	SA B (isom1) ^B^	717	MS2 [717]: 519, 475, 339; MS3 [519]: 475, 339	−	−	6.9 ± 0.5
		289, 318	Dedihydro-SA B (isom 1) ^B^	715	MS2 [715]: 313, 627, 671, 269; MS2 [313]: 179, 135	10.8 ± 0.1	−	−
20	8.5	289, 337	Chrysoeriol-*O*-glc ^B^	461	MS2 [461]: 299, 284; MS3 [299]: 284	D	D	−
21	9.0	228, 282, 331	Apigenin-*O*-glc (isom 2) ^B^	431	MS2 [431]: 269; MS3 [269]: 225, 149, 117, 183, 167, 199	D	−	−
22	9.0	252, 267, 342	Chrysoeriol-*O*-rut ^B^	607	MS2 [607]: 299, 284 ; MS3 [299]: 284	−	−	D
23	9.1	267, 333	Apigenin-*O*-glcA ^B^	445	MS2 [445]: 269, 175	2.1 ± 0.03	8.3 ± 0.05	1.97 ± 0.1
24	9.6	287, 325	RA ^A^	359	MS2 [359]: 161, 179, 197, 223	55.8 ± 2.8	40.2 ± 0.9	43.2 ± 3.2
25	9.8	287, 311	3′-*O*-(8″-Z-Caffeoyl) RA (isom 1) ^B^	537	MS2 [537]: 493, 515, 375, 357, 339, 313, 197	D	D	D
26	9.0	289, 319	Dedihydro- SA B (isom 2) ^B^	715	MS2 [715]: 313, 671, 627, 269	D	−	−
27	10.1	288, 326	SA B (isom 2) ^B^	717	MS2 [717]: 519, 357, 555 MS3 [519]: 357, 475, 295	D	D	D
		287, 324	SA K ^B^	555	MS2 [555]: 493, 357, 393, 313; MS3 [493]: 359, 313, 161	D	10.5 ± 0.1	−
28	10.7	290, 323	3′-*O*-(8″-Z-Caffeoyl) RA (isom 2) ^B^	537	MS2 [537]: 493, 359; MS3 [493]: 359, 313, 295, 161	12.0 ± 0.2	D	D
29	12.2	288, 322	Caffeoyl RA (isom 1) ^B^	537	MS2 [537]: 375, 493, 359, 519	10.5 ± 0.06	D	D
30	12.5	287, 328	Caffeoyl RA (isom 2) ^B^	537	MS2 [537]: 439, 519, 357, 493, 323, 197	4.2 ± 0.1	−	−
31	12.8	288, 323	Caffeoyl RA (isom 3) ^B^	537	MS2 [537]: 519, 359, 357, 339, 235, 493; MS3 [519]: 357	−	D	−
32	13.3	287, 323	3′-*O*-(8″-Z-Caffeoyl) RA (isom 3) ^B^	537	MS2 [537]: 493, 375, 359; MS3 [493]: 359, 197	D	D	D
			Phenolic compounds groups		*Caffeic acid and derivatives*	97.6 ± 2.6 ^a^	57.1 ± 1.3 ^b^	50.0 ± 3.8 ^c^
					*Flavones*	22.0 ± 0.3 ^a^	73.3 ± 1.0 ^b^	32.2 ± 2.0 ^c^
					*Flavonols*	2.3 ± 0.1 ^a^	3.6 ± 0.04 ^b^	1.1 ± 0.1 ^c^
					*Flavanones*	1.9 ± 0.01	−	−
					Total	123.9 ± 2.8 ^a^	134.0 ± 2.4 ^b^	83.4 ± 5.8 ^c^

*T. h-b*: *Thymus herba-barona*; *T. pseud*: *Thymus pseudolanuginosus*; *T. caes: Thymus caespititius*; D: detected; RT: retention time; CQA: caffeoylquinic acid; Der: derivative; Glc: glucoside; GlucA: glucuronide; Hex: hexoside; isom: isomer; RA: rosmarinic acid; Rut: rutinoside; SA: salvianolic acid; ^A^ compound identification was based on comparison to standard; ^B^ compound identification was based on interpretation of UV spectral and MS data, plus comparison to literature; Mean values ± standard deviations of three independent assays; Statistical analysis was performed by one-way ANOVA (Tukey’s test). In each row, different letters (a–c) stand for significant statistical different data (*p* < 0.05).

**Table 3:** MIC (mg/mL) and MBC (mg/mL) of plant extracts and nisin (mg/mL) against selected test pathogens.

Bacteria	*T. herba-barona*		*T. pseudolanuginosus*		*T. caespititius*		Nisin	
	MBC	MIC	MBC	MIC	MBC	MIC	MBC	MIC
*Salmonella typhimurium*	5.0	5.0	>6.5	6.5	>7.0	7.0	0.5	0.5
*Staphylococcus epidermidis*	5.0	5.0	6.5	6.5	7.0	3.5	<0.03	<0.03
*Staphylococcus aureus*	0.6	0.6	1.6	1.6	3.5	3.5	0.25	0.25
*Escherichia coli*	>5.0	5.0	>6.5	6.5	7.0	7.0	0.5	0.5
*Pseudomonas aeruginosa*	>5.0	5.0	>6.5	6.5	>7.0	7.0	1.0	0.5

Mean values; MIC: minimum inhibitory concentration; MBC: minimum bactericidal concentration.

## Supplementary Materials

Supplementary materials can be found at www.mdpi.com/1422-0067/18/9/1879/s1.
