# Supplementary material for: Health-Promoting Effects of Thymus herba-barona, Thymus pseudolanuginosus, and Thymus caespititius Decoctions
Source: Int J Mol Sci. 2017 Aug 31;18(9):1879. doi: 10.3390/ijms18091879 (PMC5618528; doi:10.3390/ijms18091879)
Supplement: Supplementary file 1 [file ijms-18-01879-s001.pdf]

Supplementary Material S1.

Table 1. Linearity, LOD and LOQ of four standard compounds used as references.

**Standard Compound**	**Range concentration** (μg/mL)	**n**[a]	**Slope** [b] (area counts/mg)	**Intercept** [b] (area counts /mg)	R^2	**LOD** (μg/mL)	**LOQ** (μg/mL)
L-7O-G	1 - 50	6	$204(\pm1) \times 10^5$	$-8(\pm3) \times 10^3$	0.9996	1.6	4.9
RA	26 - 517	6	$194(\pm4) \times 10^5$	$-1(\pm1) \times 10^5$	0.9970	52.1	158.0
A-7O-G	1 - 50	6	$189(\pm2) \times 10^5$	$-4(\pm3) \times 10^3$	0.9994	2.1	6.3
E-7O-G	1 - 100	6	$149(\pm1) \times 10^5$	$4(\pm41) \times 10^2$	0.9997	3.1	9.2

LOD and LOQ were defined as 3.3 and 10 times the value of the regression error divided by the slope, respectively; L-7O-G: luteolin-7-*O*-glucoside; RA: rosmarinic acid; A-7O-G: apigenin-7-*O*-glucoside; E-7O-G: eriodictyol-7-*O*-glucoside; [a] Number of points used for the regression of standard solutions. Injections were done in triplicate; [b] The standard deviation in the slope and intercept of the regression line is shown in parentheses.
